# Long-Distance Movements of Feral Cats in Semi-Arid South Australia and Implications for Conservation Management

**DOI:** 10.3390/ani11113125

**Published:** 2021-10-31

**Authors:** Jeroen Jansen, Hugh McGregor, Geoff Axford, Abbey T. Dean, Sebastien Comte, Chris N. Johnson, Katherine E. Moseby, Robert Brandle, David E. Peacock, Menna E. Jones

**Affiliations:** 1 School of Natural Sciences, University of Tasmania, Private Bag 55, Hobart, TAS 7001, Australia; hughmcgr@gmail.com (H.M.); Abbeytdean@gmail.com (A.T.D.); c.n.johnson@utas.edu.au (C.N.J.); menna.jones@utas.edu.au (M.E.J.); 2 Arid Recovery, Roxby Downs, SA 5725, Australia; 3 National Environmental Science Program, Threatened Species Recovery Hub, University of Queensland, Brisbane, QLD 4067, Australia; 4 Department for Environment and Water, Port Augusta, SA 5700, Australia; Geoff.Axford@sa.gov.au (G.A.); Robert.Brandle@sa.gov.au (R.B.); 5 Vertebrate Pest Research Unit, NSW Department of Primary Industries, Orange, NSW 2800, Australia; sebastien.comte@dpi.nsw.gov.au; 6 Centre for Ecosystem Science, University of New South Wales, Kensington, NSW 2052, Australia; katherine.moseby@adelaide.edu.au; 7 School of Animal and Veterinary Sciences, University of Adelaide, Roseworthy, SA 5371, Australia; david.peacock@adelaide.edu.au

**Keywords:** *Felis catus*, introduced predator, invasive species management, reinvasion, space use, landscape, relocation, telemetry, home range

## Abstract

**Simple Summary:**

To efficiently control invasive animals, it is vital to have knowledge about their behaviour, their movements and how they use the landscape. Unusual behaviour is normally excluded from datasets, as it is considered to be an outlier that may distort analyses. In our study, we present movement data from feral cats in the arid and semi-arid zones of Australia. Feral cats are a serious problem to the native wildlife of Australia and in many parts of the world. Cats are known to show fidelity to geographic areas and may defend them against other cats. Until now, research has focused on these areas, home ranges or territories, that feral cats need to survive and reproduce. We argue that a part of their movement behaviour, large journeys away from the area they normally use, has been overlooked and has been considered to be unusual behaviour. We explain why we think that this is the case and present examples from other studies additional to our data set to show that these long-distance movements are a regular occurrence. To achieve a better protection of native wildlife from predation by feral cats, we believe that these long-distance movements should be considered as part of the normal behaviour of feral cats when planning cat control operations.

**Abstract:**

Movements that extend beyond the usual space use of an animal have been documented in a range of species and are particularly prevalent in arid areas. We present long-distance movement data on five feral cats (*Felis catus*) GPS/VHF-collared during two different research projects in arid and semi-arid Australia. We compare these movements with data from other feral cat studies. Over a study period of three months in the Ikara-Flinders Ranges National Park, 4 out of 19 collared cats moved to sites that were 31, 41, 53 and 86 km away. Three of the cats were males, one female; their weight was between 2.1 and 4.1 kg. Two of the cats returned to the area of capture after three and six weeks. During the other study at Arid Recovery, one collared male cat (2.5 kg) was relocated after two years at a distance of 369 km from the area of collar deployment to the relocation area. The movements occurred following three years of record low rainfall. Our results build on the knowledge base of long-distance movements of feral cats reported at arid study sites and support the assertion that landscape-scale cat control programs in arid and semi-arid areas need to be of a sufficiently large scale to avoid rapid reinvasion and to effectively reduce cat density. Locally, cat control strategies need to be adjusted to improve coverage of areas highly used by cats to increase the efficiency of control operations.

## 1. Introduction

Knowledge on the incidence and magnitude of large-range movements is important in understanding the spatial behaviour, potential impacts and hence management of wildlife. This is especially the case for significant invasive pest species [1], such as feral cats (*Felis catus*) in Australia [2]. The development of GPS collars has greatly improved our understanding of the capacity of wild animals for long-distance movement. For example, a study of foxes (*Vulpes vulpes*) in Norway and Sweden found that six individuals travelled distances between 132 and 1036 km in less than a month [3], whereas an older eight-year tag return study of the same species in North Dakota (USA) recorded a maximum displacement of 302 km based on the recoveries of 363 of 854 tagged foxes [4]. Individual movements of wildlife are often divided into home-range movements [5], dispersal [6], exploration [7] and migration [8]. The home range is defined as the area used by the animal for routine activities and to gather resources needed for survival and reproduction [9]. Its species-typical size is related to body mass and trophic position [10]. Home ranges are dynamic measures of movements over time and can therefore vary with the timescales over which they are studied. In feral cats, home range size has a high intraspecific variability (see e.g., [11,12]) and can change with season [13,14], sex [15], resource availability [16,17], and climate [18]. The distinctions between movements within the home range and exploration or dispersal beyond it are therefore relative to the considered timescale [19]. Space use is now more often represented as utilisation distribution (UD), defined as the probability of an animal using a given location [20,21,22].

Knowledge of the UD of pest animals is crucial for understanding the efficacy of pest management-based open landscape conservation programs. An example of such a program is Bounceback [23], which operates across public and private conservation reserves and pastoral leases in the semi-arid ranges of South Australia, including the Ikara-Flinders Ranges National Park (IFRNP). This 938 km^2^ semi-arid reserve in northern South Australia and many neighbouring properties have been fox-baited since 1992, including aerial baiting annually since 2007, as part of a program to achieve landscape-scale recovery of native fauna [24]. Since 2017, an annual experimental aerial-baiting program using the Eradicat^®^ 1080 cat bait (Australian Pesticides & Veterinary Medicines Authority research permit PER84037) has been undertaken over a 650 km^2^ area incorporating the western half of IFRNP and most of the Arkaba Conservancy (ARKC), a private conservation property adjacent to and south of Wilpena Pound at the southern edge of the IFRNP [25]. These control efforts over a vast area have enabled successful faunal recovery and reintroduction projects [26,27]. However, even on this scale the threats associated with the long-distance movements of feral cats, as previously reported [14,28,29,30], exceed the scale of the cat management area.

Documenting dispersal or long-distance movement of feral cats is difficult. Even though new technology that transmits animal locations directly to satellites (e.g., ARGOS) is available, this significantly increases the costs of collars [31]. Therefore, most datasets on cat movements have been based on telemetry collars that are re-located, generally manually, using a VHF beacon. These typically have a detection range of 1–10 km. Even if the collar has a store-on-board GPS unit, it still must be located using the VHF beacon for the data to be retrieved. If the beacon can no longer be detected, it is impossible to know whether the cat moved beyond the scanned range, or if the collar failed. Failure to retrieve collars is common in animal-tracking studies [32], and searching for collars by methods such as tracking with small aircraft [33] increases study costs tremendously. Recovery of data on long-distance movement therefore often depends on animals returning to the study area [34,35]. The published datasets on animal movements could therefore underestimate the true mobility of feral cats. 

This study reports on the long-distance movements of feral cats collared with GPS/VHF collars for a study of home range behaviour and habitat preference in the semi-arid region of South Australia over a three-month period and a relocation of a cat two-years after it was collared, including the movement characteristics and individual attributes of cats that undertook these long-range movements. We discuss the implications for regional control programs, as movements went across administrative boundaries and out of pest-controlled baiting areas.

## 2. Materials and Methods

**Figure 1:**
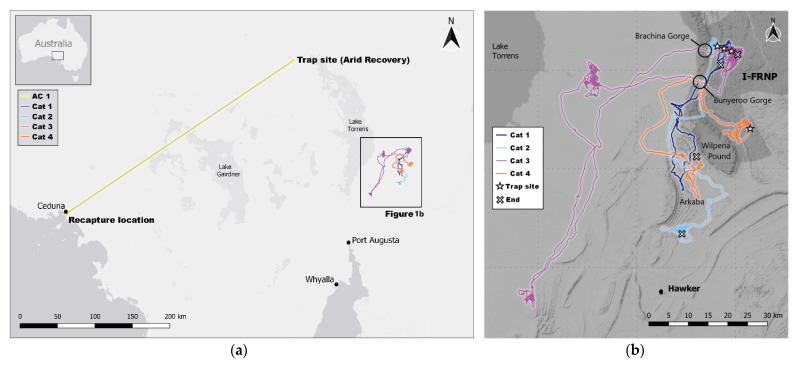
(**a**) Movement of feral cat (AC1) collared at Arid Recovery, South Australia, with trap site and site of last capture. The location of (**b**) is indicated with the box; (**b**) Movement tracks of the four feral cats collared in the Ikara-Flinders Ranges National Park (IFRNP), South Australia, with trap sites (☆) and site of last capture/death (X). Dark blue = Cat 1; Light blue = Cat 2; Purple = Cat 3; Orange = Cat 4. The location of Hawker township, Arkaba Conservancy, Wilpena Pound and the two gorges incising the Heyson Range are shown. The National Park is highlighted in dark grey. Base image: (**a**) ESRI Grey/QGIS; (**b**) Stamen/QGIS [37]. Map created using the Free and Open Source QGIS.

The feral cats were captured in the IFRNP (31.25° S, 138.42° E) and the adjacent Arkaba Conservancy (ARKC; 31.73° S, 138.50° E), and at the Arid Recovery Reserve (AR; 30.25° S, 136.94° E), all located in semi-arid and arid South Australia (Figure 1a,b). Temperatures reach a daily average maximum of 25.6–34.2 °C in the summer months (October to March), reducing to 16.0–25.4 °C in winter (June to August). Rainfall is low (300–400 mm north–south around IFRNP and ARKC, and 170 mm near AR), and is sporadic, with a winter bias starting in April [36]. The years of 2017 to 2020 are the driest period on record.

Between 25 February and 5 May 2020, 19 feral cats were captured in the IFRNP and adjacent ARKC using large cage traps baited with rabbit meat. Cats were transferred to a cotton bag, weighed, sexed, fitted with a Telemetry Solutions 4000ER GPS/VHF collar (Table 1), and released at the point of capture. The GPS trackers on the collars were programmed to record one location fix every 15 min. The position of the cats was monitored daily with a magnetic base omni-antenna attached to the roof of a car and via telemetry triangulation from elevated positions such as hilltops with a 5 element 150 MHz folding antenna (Advanced Telemetry Systems). The first few months of the study were affected by COVID-19 restrictions not allowing the use of aircraft for tracking, but once these eased in mid-May 2020, cats were additionally located using weekly flights in a Cessna 172 plane with a Civil Aviation Safety Authority-approved VHF antenna (Faunatech) affixed to the wing strut. The flight path followed parallel lines across and extending beyond the entire study area (about 1000 km^2^) while searching for all collar frequencies. Later, the area searched was extended to an area of around 9000 km^2^, bounded by Port Augusta, located 160 km to the south, and Parachilna, 60 km to the north of the study area, to search for missing collars. Once a month, animals were tracked to their location, at which point their stored GPS fixes were remotely downloaded by Bluetooth using a base station (Telemetry Solutions). 

Cat “AC1” was captured during research conducted around AR, being trapped on the outside northern edge of the reserve 40 km north of Roxby Downs and 70 km south of Lake Eyre (Figure 1a). AC1 was caught on 19 February 2017 in a cage trap baited with cat urine, and fitted with a collar including a GPS data-logger and a VHF transmitter (Robin tag, model G10 UltraLITE, Advanced Telemetry Solutions, Isanti, MN, USA) weighing 65 g. The collar was programmed to collect high-frequency fixes at 150 s intervals. After capture, weighing and collar-fitting, the cat was released at the point of capture. On release of the cat, the VHF beacon was tested and confirmed as working.

All cats collared in both studies (IFRNP and AR) were euthanised at the end of the study. This is in accordance with the pest animal regulations of South Australia.

Location data were cleaned using R version 4.0.3 [38] and R studio version 1.3.1093 [39] and then mapped using QGIS version 3.6.0 [37]. GPS fixes were discarded when less than 3 satellites were used to establish the location, or when 3 or more satellites were used but the horizontal dilution of precision (Hdop) was higher than 7 and only a 2D fix could be achieved (that is, lacking altitude, so that the accuracy of the horizontal position is compromised) [40]. Remaining outlying location data points were removed by calculating the speed between consecutive locations. We used a threshold of 20 km/h over a period of 15 min between fixes. This method cuts off extreme speeds indicating that the position is wrong but allows for unusual behaviour to remain in the dataset. GPS fixes recorded when a cat was in a trap were deleted. 

We compiled previously published data on maximum values for feral cat movements away from their site of capture, the maximum values for home ranges and the method of calculating the home range (Table 2). Most studies on this list consider movements within an area of 10,000 ha to be normal home range movements. A movement in our study was therefore considered a long-range movement if the cat moved further than 10 km from the site of capture.

We calculated the following metrics for each cat that made at least one long-range movement: Total distance travelled was the sum of the linear distances between successive location fixes from the collaring location to the location of collar retrieval (Table 1). The longest movement diameter was the longest linear distance between any two location fixes of a given cat. Total daily movement distance (ddist) was calculated by summing the distances between all consecutive relocations for an individual across a day. Then, the median, mean and standard deviation of ddist was calculated. Deviation (dev) from the median daily movement was calculated using the median of all daily movement distances travelled by each individual cat (medmov) and its daily movement distance (dev):

dev = (ddist − medmov)/medmov ∗ 100,(1)

The number and proportions of GPS points within and outside of the conservation properties of IFRNP and ARKC were analysed to evaluate movement out of conservation-managed areas. Movements within the conservation properties were distinguished between points that fell into the aerially baited zones (50 baits/km^2^) and non-aerially baited areas (Eradicat^®^ baited, non-baited within National Park, non-baited within ARKC, outside of IFRNP and ARK (non-baited)). Buffer zones around roads and tracks in the IFRNP and ARK were hand-baited to a low density of about 10 baits/km^2^ and were counted in the non-baited areas [11].

Minimum Convex Polygons (MCP) and Kernel Density Estimation (KDE) with href (reference bandwidth) are classically used to estimate home-range sizes [45,46]. MCP and KDE(href) are included here to allow comparisons with earlier publications. Two additional methods were used to provide more accurate estimates of the cats UD: the KDE using least square cross validation (lscv) and biased random bridges (BRB), as they exclude unexplored areas [21,22,47]. The diffusion coefficient for BRB was calculated, setting the maximum duration allowed for a step built by successive relocations (Tmax) to 43,200 s and the minimum distance between successive relocations (Lmin) to 100 m. The minimum smoothing parameter was set to 100 m, and tau to 288 s. MCP, KDE(href), KDE(lscv) and BRB were calculated using the Adehabitat package [48].

## 3. Results

Of the 19 adult cats (5 female, 14 male) that were radio-collared in IFRNP between 25 February and 5 May 2020, the collars on 4 cats recorded GPS points for less than 20 days. The fate of two collared cats (1.9 kg and 5.0 kg males) remains unknown as they could not be relocated despite expanding our search area from the study site to an area of around 9000 km^2^ and the use of aircraft. Only the data for the remaining 13 cats (4 female = 30.8%, 9 male = 69.3%) is considered for this study. Their collars recorded GPS points for a period of between 41 and 119 days. Four of the cats (Cat 1–4) moved further than 10 km from their site of collaring (1 female = 25%, 3 male = 75%). The remaining nine cats (Cat 5–13) stayed within 10 km of their trap site, except for one walk of 13.9 km from the trap site undertaken by Cat 13, returning after a day.

Of the 11 cats collared before 15th of March that returned data for more than 20 days, only two remained in the same area that they were trapped (four moved away, two could not be relocated, one was euthanised because it was emaciated and two died, presumably from starvation). In contrast, none of the seven cats collared after 15th of March moved away from the area where they were trapped. One of the cats died of presumed predation and was found in an open area partially eaten. Another one died of unknown causes after 41 days and was found in the entrance of a rabbit warren. Of the nine collared cats still alive during the annual aerial Eradicat^®^ baiting in May 2020, five survived and were euthanised one month later. The average daily distance they walked was 6.5 ± 3.3 km (median = 5.3 km).

**Figure 2:**
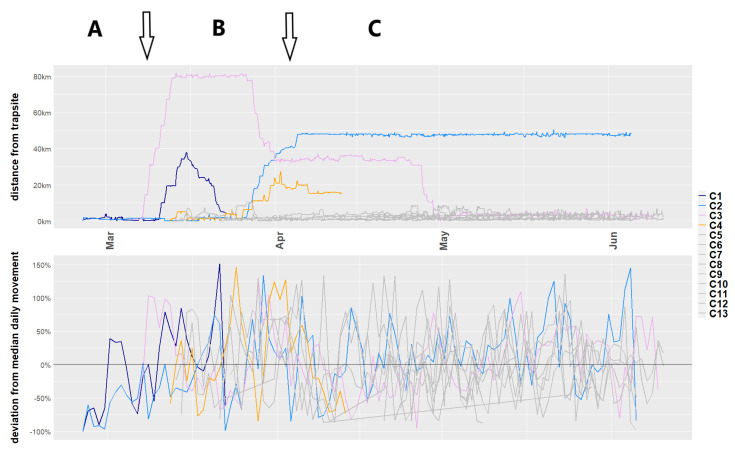
Movement summary over time (February to June 2020) of the feral cats captured in the IFRNP (Dark Blue = Cat 1; Light blue = Cat 2; Purple = Cat 3; Orange = Cat 4). Arrows indicating change in movement behaviour between sedentary (A and C) and long-distance movements (B).

Of the four cats that undertook long-range movements from the IFRNP, three were male with bodyweights of 2.1 (Cat 1), 2.7 (Cat 2) and 4.1 kg (Cat 3), all trapped in the north-western part of the IFRNP, and one was a female weighing 2.4 kg (Cat 4), trapped in the south-western part (Figure 1b and Table 1). Cat 3 left the area of capture after one day whereas Cat 1, 2 and 4 remained for 17, 33 and 14 days in the area before moving more than 10 km from their site of capture. The long-range movements of Cats 1–4 occurred between 7 March and 30 April 2021 (Figure 2).

Cat 1 was trapped in an aerial baiting buffer zone, 250 m from the border of the baited zone. It left the initial area after 17 days and moved for 11 days, heading south for five days before returning towards the area of capture and dying within the baited zone (cause unknown) 4.5 km from where it was collared. It was found in a resting position below the roots of a tree.

Cat 2 was trapped in an aerial baiting buffer zone, 100 m from the border of the baited zone. It stayed in the area of capture for 33 days and moved south for seven days after that to remain in the south of ARKC for nine weeks (Figure 1b). It was found dead 1400 m outside of the baited zone shortly after the aerial Eradicat^®^ baiting and with physical signs similar to cats that had died after ingesting a bait. The GPS tracks showed that it had been in the baited zone shortly before dying.

Cat 3 was trapped in an aerial baiting buffer zone, 200 m from the border of the baited zone. It left the area of capture a day after getting trapped. It went south-west, spending 14 days in an area 34 km west of the Hawker township, then back north to near Lake Torrens, where it spent 25 days before heading east and returning to where it was first trapped and collared on IFRNP. It remained in that area for another three months before it was captured and euthanised at the end of the study.

Cat 4 was trapped within the baited zone in the south-western part of the IFRNP. It stayed in the area of capture for 14 days before traveling to ARKC, arriving after seven days and remaining for another 11 days before dying in a resting position in a tree hollow 80 m from the border but within the baited zone, cause unknown.

The average movement of Cats 1–4 was between 6.7 ± 3.6 km and almost 10 km (9.9 ± 4.7 km) per day (see Table 1), covering maximal geographic distances of between 30 and 85 km from the most distant points over periods of 8 to 53 days. Most of the long-distance movements to other areas by the cats collared in the IFRNP occurred in March up to mid-April (Figure 2), apart from one long-range movement by Cat 3 at the end of April. Even though the distance from the trap-site increased for all cats during their long-range movements, the deviation from the median of the daily distance over the survey period remained similar to the cats that remained in the area of capture (see Figure 2). 

**Figure 3:**
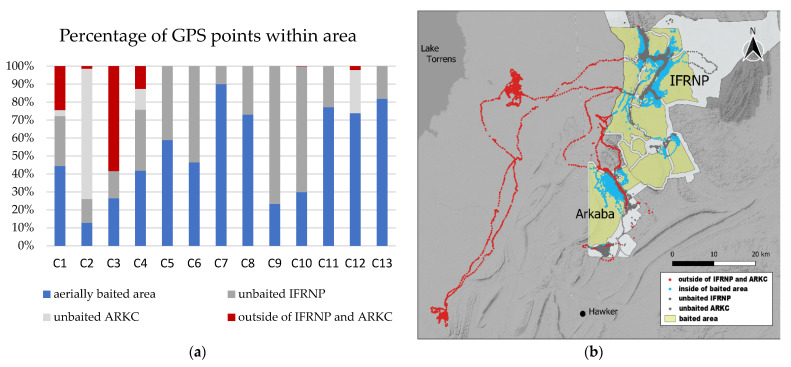
(**a**) Proportions of GPS points of the collared feral cats that fell within the aerially baited zone, the Ikara-Flinders Ranges National Park (IFRNP), Arkaba Conservancy (ARKC) or outside of the National Park and Arkaba; (**b**) GPS points of the feral cats that were collared in 2020 falling outside (red) of the conservation areas IFRNP and Arkaba and within (grey). GPS points that were in the aerially Eradicat^®^-baited area are marked in blue. The National Park and Arkaba are highlighted in white. Base image: Stamen/QGIS [37]. Map created using the Free and Open Source QGIS.

The area covered by the movements of Cats 1–4 were between 23,341 and 157,834 ha (calculated as 95% MCP; Table 1). Excluding unexplored areas by calculating KDE(lscv) and BRB, the area covered by their movements was between 1634 and 19,171 ha (95% KDE(lscv)) and 2815 and 6575 ha (95% BRB). The percentage of GPS points within the aerially baited zone varied between 12.7 and 90% for the different individual cats (see Table 3, Figure 3). Seven of the 13 cats had less than 50% of their GPS points falling within the aerially baited zone. The only cats that had a significant proportion of GPS points outside of the boundaries of the conservation areas IFRNP and ARK were Cats 1, 3 and 4, during their long-distance movements.

The four IFRNP cats remained mostly in the same habitat types and geological layers: open, soft-soiled areas with sparse vegetation and high European rabbit (*Oryctolagus cuniculus*) densities [49]. Only Cats 1 and 4 spent short periods of hours to a few days, during their long-distance movement, in other habitat types and geological layers, such as forested areas with harder soils and lower rabbit densities. Both of these cats died, well before the Eradicat^®^ baiting, and as their bodies were found intact in a resting position in a sheltered spot, it is presumed they did not die of predation.

It is of note that three of the IFRNP collared cats used the two main gorges (Brachina and Bunyeroo Gorge) through the prominent north–south oriented Heysen Range (highest peak 1189 m), either partially or fully to access the flatter region west of the ranges before moving southwards (Figure 1). Only Cat 2 did not use the main gorges, but crossed the rugged Heysen Range at a high saddle between two peaks at the northern end of the Wilpena Pound wall. The tracks of all of the cats were directional, following the north–south orientation of the major ranges. Cat 3, during its return north back towards the IFRNP at the end of April 2020, crossed the Heyson Range, walking from a distance of about 22 km straight towards and through the Bunyeroo Gorge.

The cat collared at AR (AC1) was a 2.6 kg male in average condition (as determined from the amount of muscle between its hip bones). It was not detected again after the initial release in February 2017 at AR, despite scanning from all prominent hills in the study area using a five-prong long-range antenna, and two flights in a light aircraft with long-range VHF antenna covering an area of 40 km in all directions from the reserve. The next record for the cat was 872 days later on 11 July 2019, when it was caught 369 km away (in a straight line) in Ceduna, South Australia by the local council as part of their control operations. It was still wearing its collar. The cat was in excellent condition and there were no signs that the collar was negatively affecting it, despite having nearly doubled its weight to five kgs. The movement track of AC1 between the site of capture and recapture is unknown. The GPS unit only recorded three days of movement before premature failure, during which the cat had remained within three kilometres of the site of initial capture.

## 4. Discussion

Our results highlight the potential high mobility of feral cats in semi-arid and arid Australia, adding to the few published records of long-distance movements by feral cats in Australia (see Table 2). The combined records indicate that large displacements of more than 10 km should be considered a potentially regular occurrence for feral cats in semi-arid and arid areas, rather than a rare event. Our study advances earlier records in that we report the maximum linear distance between locations. Of the 13 feral cats GPS-collared for more than 20 days in the IFRNP in 2020, 4 (75% male and 25% female) showed long distance movements of more than 10 km, which in this small sample size is a bit lower than the female sex ratio of the total captured cats (68.2% male and 30.8% female). The expectation was that all of the collared cats would remain around the area that they were trapped in. After leaving the area of capture, these four cats that undertook long-range movements could only be found by investing the time, money and effort to repeatedly search an extensive area of approximately 9000 km^2^ by plane. Another two of the 13 collars deployed in 2020 (attached to 1.93 kg and 5 kg male cats) could not be relocated despite the intense searching effort. As the fate of these animals cannot be determined, the possibility remains that these cats also conducted long-distance movements beyond the extensive area searched by aircraft. The low probability recapture of AC1 after two years of no detection and its subsequent capture as part of a localised control program at a site in a straight-line distance of about 370 km away, is evidence that not all lost VHF signals can be attributed to collar failure. Cats are easily capable of moving way beyond the area searched during most surveys. A high number of collars placed on feral cats are not retrieved [32]. Undetected long-distance movements might be an explanation for why some of the collars were not relocated. Emerging technologies, such as remote submission of collar location data via satellite [14], might eliminate the relocation problem and will improve our understanding of the long-distance movements of feral cats. The initial higher costs of satellite collars need to be weighed against the costs of aerial search and the potential loss of GPS-VHF collars and data.

The size of the areas traversed by Cats 1–4 in this study exceed most previously published maximum values of 95% MCP in feral cats of 131.98 km^2^ and 229.0 km^2^ [11,30]. Only the most recent study by Roshier and Carter [14], which used satellite technology, exceeds the areas covered when calculated using MCP including all GPS points. Newer methods of calculating home range such as KDE (lscv) and BRB exclude un-utilised areas when estimating the UD. Using these methods to calculate space use, the size of the areas used by Cats 1–4 have smaller values, but still exceed older records using 95% MCP. We are still only beginning to understand the movement patterns of feral cats in semi-arid and arid areas. Long-distance displacements need to be evaluated differently. Unusual behaviours are often removed from the data set during home range analysis as these are viewed as outliers (e.g., see [11,30]). This seems a reasonable approach during data handling as extreme movements are unusual and could be considered as location errors or dispersal rather than within-home range movements; in the latter case, natal dispersal movements would not contribute to an understanding of home range. For conservation purposes, however, large displacements are of interest, because these determine how fast feral cats can reinvade after management efforts to suppress populations in a defined area. 

Numerous control programs in open landscapes have documented little subsequent change in the number of cats detected despite high levels of cat mortality [42,50,51,52,53]. One plausible explanation for these observations is that there is rapid reinvasion of individuals from surrounding areas, as reported from studies of similar meso-predators [54,55,56], including cats [11,42]. Considering the distances travelled and areas traversed by the cats in this study (Table 1), and other previously recorded long-distance displacements (Table 2), it is obvious that even the extensive areas covered by aerial baiting of cats in our study (650 km^2^ of aerial Eradicat^®^ baiting in the IFRNP and ARKC) are likely to be too small to prevent rapid reinvasion into areas where cat control is undertaken. Cats in our study moved vast distances, up to 369 km in a straight line, demonstrating how the scale of movement of some feral cats could potentially undermine control efforts conducted at both local and large scales. The importance of coordinating conservation management cat control in light of the potential of cats to move long distances is highlighted by Cat 2, which was collared in the IFRNP and likely died of bait uptake in the extended baiting area after it moved to the neighbouring conservation property of Arkaba. 

The motivations behind long-range movements in feral cats have been hypothesised to be drought, i.e., searching for food [28], dispersal [57], exploration [11] or occupation of vacant territories [43]. There is a trade-off between moving and improving the chances of survival and reproduction, or residing and adjusting to difficult conditions [8]. Understanding the patterns of these large-scale movements, and their associated demographic and environmental factors, can provide important insights into the drivers of the variation in the predation impact of feral cats [58,59]. This knowledge also highlights the potential for severe predatory impact on small, geographically restricted populations of vulnerable prey species. Cats clearly have the capacity and inclination to undertake long-distance movements of hundreds of kilometres in a period of weeks [42]. The 13 cats in our study showed consistent daily movements averaging 6.5 km/day over extended periods of time, indicating that this is a common occurrence in cats in semi-arid South Australia. When travelling outside of the local area where the cats were collared and most maintained home ranges, the movement patterns changed to more directional linear movements, although the daily sum of movement did not differ much from their more localised movements. The outcome of these bursts of linear movement is a rapid geographic displacement, visible especially in the movement of Cat 3 in mid-March (see Figure 2). This directionality suggests that these movements were intentional, not random [60]. The apparently intentional movement through the gorges, using these to cross high and rugged ranges, suggests they might have a knowledge of the landscape from previous long-distance movements. The body weight of Cat 3 (4.1 kg) indicates this is obviously a mature cat and so its movements are unlikely to be natal dispersal. 

Nutritional stress was suggested to be a primary motivation for the long-distance movements in cats documented by Edwards et al. [29]. The survival and breeding of the major prey of cats, which is rabbits in this semi-arid environment, is reduced in summer and during longer droughts [61,62]. The dates of the long-distance movements occurred between late February and early April, which is at the end of the hot, dry summer in South Australia, when the rabbits are at their lowest abundance in the year [63,64,65]. Subsequent establishment of new territories coincides with the usual period of onset of winter rains in early to mid-April, when rabbits recommence breeding, and with young rabbits emerging shortly after [66]. The movement patterns of Cats 2 and 3 are consistent with the seasonal recovery of rabbit prey; they settled in early April, albeit in a different geographic area from where they were trapped. The return of Cat 3, the large male, to the IFRNP in mid-April suggests that this cat returned to a known predictable food supply. We can only infer the movement response of these cats in relation to the general seasonal dynamics of rabbit populations and knowledge that rabbit density varies widely across the landscapes traversed by these cats. As the cats in the IFRNP were collared for a maximum of 128 days, we cannot be sure whether this is the case, even though other studies of feral cats suggest that there are differences in prey selection over seasons [58] and that seasonal changes in home range size connected to landscape productivity exist [17].

Long-distance movements of cats in the Kimberley region of Western Australia reinforce food resources as a key driver underlying long-distance movement behaviour. McGregor et al. [44] reported on long-distance extra-territorial journeys by larger adult male feral cats (3.2–5.1 kg) to hunt on fire scars following intense burns of up to 12 km distance, with subsequent returning to their initial home range. While these records are of a shorter distance than we report and are in response to discreet events, they are also apparently in response to enhanced food resources—in these cases improved hunting success on intense recent burn scars. These extra-territorial excursions lasted about one month, by which time the cats and other predators might have depleted the available prey on the fire scar. The Kimberley records are only of large male cats, the demographic population class which is consistently identified as having a high impact on reintroduced and declining small populations of native mammals [67]. Even though the longest and furthest movement was made by the largest male cat, our South Australian records involve much greater distances than the Kimberley records, a variety of sizes and sexes, and not all cats returned to their original location. This indicates that the reasons for movement might be either different for every cat or affect all cats in the same way. Reduced prey availability leads to higher mortality in feral cats [65]. This is particularly prevalent at the end of summer and in drought conditions, when the harsh conditions lower the prey and feral cat populations in the area. Consequently, there will be more vacant territories once prey availability recovers following rain. The movements of the cats could therefore be triggered by the intention of occupying vacant territories or potentially establish a better territory with more resources. As Cat 3 was the only large adult cat at the beginning of the study, we cannot exclude natal dispersal as an explanation for the long-distance movements of the other cats, although at >2 kg they would be considered adults.

The long-distance cat movements we recorded in the IFRNP and ARKC reflect both the north–south alignment of the Flinders Ranges and the increasing north to south rainfall and productivity gradient in South Australia [36]. The Heysen and ABC Ranges in the IFRNP appear to channel the movement of cats generally in a north–south direction. As these relatively narrow ranges are high, rugged and extensive, they influence long-distance as well as local home-range scale movements. To cross the high and rugged Heysen Range, cats used the major gorges and lower saddles that cut through the ranges. The timing of the long-distance movements reflects both geographic and seasonal rainfall patterns. The cats that moved long distances went in a generally southerly direction, indicating that the cats may consider this general rainfall and productivity pattern in a search for better food resources. Similarly, a southerly movement was found for all cats that exhibited long range movements from Arid Recovery [11]. The northerly return movements to the IFRNP in April coincided with the prospective seasonal increase in availability of prey, particularly young rabbits, as rabbit breeding recommences once the weather cools and rains start from April onwards. This movement response is especially visible in the largest of the four cats (Cat 3), which showed a very directional movement between three distinct areas of foraging (see Figure 1b and Figure 2). The cessation of long-distance movements seems to have preceded the emergence of young rabbits from warrens by weeks, suggesting that mature cats retain knowledge of the landscape, prey dynamics and timing. Future studies might be able to confirm this by collaring cats during the summer. 

The question remains of how feral cats detect distant resources and navigate to those areas [42]. The linearity of movements of the cats suggests that their long-distance movements are intentional and target areas with high productivity, such as the low, flat areas with high rabbit densities around IFRNP. These areas need to be targeted for cat control. Even though all the collared cats were trapped adjacent to or in the baited areas in the IFRNP, they spent up to 87.3% of the monitored time in non-baited or hand-baited buffer areas that have low bait density because they are around publicly accessible features such as roads (see Table 3, Figure 3). The lower bait density in these buffer zones combined with the high availability of rabbits as prey might account for the lower mortality during the baiting event in this area [68]. Our results highlight that localized cat management may not be sufficient to account for the scope of cat movement. 

## 5. Conclusions

The long-distance movements of feral cats described in this study present a different aspect of movement behaviour which could have important implications for small populations of threatened fauna. These records highlight the great distances that cats are capable of moving in hot and semi-arid environments, such as those found in South Australia. Our study shows that wide-ranging cats include both sexes and a range of body weights, and a potential range of motivations that might drive their long-distance movements. Whether long-distance movements are dubbed as large within-home range movements, natal dispersal [69], extraterritorial journeys [44], shifting home ranges [14], partial nomadism [8] or exploratory walks [30] is of less importance than considering them as being a regular occurrence [70]. Our results strengthen the notion that feral cats seem highly proficient at navigating a vast semi-arid landscape and go some way to explaining why so many cat-removal operations struggle to achieve and maintain low cat abundance. Scaling conservation management to account for the mobility of feral cats is a major challenge [2]. The data indicate that the source area that could provide new immigrants into cat control areas is potentially very large. Conversely, this finding also suggests that conservation actions in one place can have far-reaching impacts. Cats moving between adjacent properties around the IFRNP and as far as Ceduna from Arid Recovery show the necessity for designing and implementing coordinated control programs at regional and geographic scales to successfully manage the impacts of cat predation within the boundaries of targeted conservation areas. When aiming for the effective control of cat predation across an area, much more extensively than considered in previous programs, cat management needs to be done across administrative boundaries and across different land-uses [71,72]. To achieve intensive feral animal control in a small area is difficult, but it becomes exponentially more costly as the area increases [73]. Therefore, when designing strategies for feral cat control, datasets highlighting high resource areas within the potential and normal range of movement of cats should be considered to better protect reintroduced or relict populations of threatened fauna [59,74]. A planned strategy focusing intensified management with a combination of control methods [51] in high food resource areas for cats, such as areas with high rabbit densities, can potentially achieve better results on a large scale. These areas will have the highest cat densities and so the control efforts will remove many cats, but this needs to be done in connection with rabbit reduction. Large-scale control requires cooperation with private landowners around conservation areas. Invasive alien predators such as cats, which are in the 100 most destructive invasive species globally, do not respect conservation boundaries. Our findings highlight their capacity to invade from surrounding areas, including from far distances, as well as the significant challenge they present to conservation managers.

## Figures and Tables

**Table 1:** Spatial, movement and fundamental metadata for the five GPS-collared feral cats that undertook long distance movements in semi-arid South Australia. Cats 1–4 were captured in the IFRNP, AC1 was captured at Arid Recovery.

	**Cat 1**	**Cat 2**	**Cat 3**	**Cat 4**	**AC1**
Sex	M	M	M	F	M
Trap date	25 February 2020	25 February 2020	6 March 2020	12 March 2020	19 February 2017
Trap site	IFRNP Brachina	IFRNP Brachina	IFRNP Brachina	IFRNP Upalinna	Arid Recovery
Trap site Easting	271,800	270,929	272,157	278,284	685,223
Trap site Northing	6,531,098	6,531,166	6,530,531	6,510,908	6,652,470
Zone	54 J	54 J	54 J	54 J	53
Weight (kg)	2.1	2.7	4.1	2.4	2.6
Collar weight (g)	80	89	90	83	65
Collar % of bodyweight	3.81	3.27	2.22	3.42	2.5
Colour	grey tabby	bright grey tabby	grey tabby	grey tabby	grey tabby
Fate	Natural death	Eradicat^®^ intake	Euthanised	Natural death	Euthanised
Last GPS movement	20 March 2020	5 June 2020	9 June 2020	10 April 2020	11 July 2019
Last location	Yanyanna track	Arkaba South	Brachina East	Arkaba North	Ceduna
Last location Easting	271,318	260,719	273,138	264,922	370,358
Last location Northing	6,526,582	6,483,663	6,530,200	6,503,602	6,442,562
Days collared	39	103	128	42	872
Final weight (kg)	-	2.8	4.3	-	5.0
Weight loss/gain (g)	-	30	235	-	2400
Median distance/day (m)	7350	6525	9375	7220	-
Mean dist./day ± SD (m)	6920 ± 4480	6702 ± 3632	9938 ± 4664	7808 ± 4593	
Total distance (km)	193.8	683.6	954.1	257.7	-
Longest diameter distance (km)	40.5	52.7	85.6	31.2	369
95% MCP (ha)	23,341	56,804	157,834	42,743	-
95% KDE href (ha)	49,841	47,348	207,727	43,514	-
95% KDE lscv (ha)	6683	1634	19,171	5095	-
95% BRB (ha)	2815	4764	6332	6575	-
50% BRB (ha)	130	100	314	88	-

**Table 2:** Spatial movement and home range of feral cats from other studies in Australia.

Locality	Distance Moved	Home Range Size	Duration Used for Calculation	Method of Calculating	Reference
Flinders Ranges (IFRNP) and Roxby Downs (AR), SA	85 km and returned (IFRNP) and 370 km (AR)	max. male (IFRNP) 157,834 ha max. female (FR) 42,743 ha	3 months (IFRNP) and 2 years (AR)	95% MCP and other	This study
Yathong Nature Reserve, NSW	8–48 and over 200 km	max. male 990 ha max. female 270 ha	3 years	MCP	[28]
Scotia Wildlife Reserve, NSW	male: 164 km and returned, female: 150 km and returned	max. male 331,351 ha max. female 196,566 ha	seasonal over 3 years	95% MCP and other	[14]
Fortescue River (Pilbara), WA	male: 130 km and returned	-	in 19 days	-	[41]
Diamantina, QLD	female cat 110 km and all 7 males ≥ 20 km	-	in 10 days	-	[42]
Kangaroo Island, SA	Aprox. 50 km	max. male 567 ha max. female 467 ha	2 years	MCP	[43]
Arid Recovery Reserve, SA	45 km in 2 days, 26 km in 3 days 35 km in 8 months and returned	max. male 13,198 ha max. female 3565 ha	2 months	95% MCP	[11]
Hamilton Downs station, NT	34 km	mean male 2210.5 ha	15–24 months	100% MCP	[29]
Kimberley, WA	30 km and returned	max. male 2006 ha	3 years	95% kernel-based	[44]
Pilbara, WA	-	max. male 20,897 ha max. female 22,904 ha	2 years	95% MCP	[30]
global	-	median male 510 ha max. female 2320 ha	-	MCP, kernel-based and other	[17]

**Table 3:** GPS points of the collared feral cats that fell within the baited zone, the Ikara-Flinders Ranges National Park (IFRNP), Arkaba Conservancy (ARKC) or outside of the National Park and Arkaba.

ID	Number of GPS Fixes	Number of GPS Fixes Within				Percentage of GPS Fixes Within			
		Baited Area	Unbaited IFRNP	Unbaited ARKC	Outside	Baited Area	Unbaited IFRNP	Unbaited ARKC	Outside
C1	1884	836	524	64	460	44.4	27.8	3.4	24.4
C2	6014	766	797	4369	82	12.7	13.3	72.7	1.4
C3	7677	2031	1163	0	4483	26.5	15.2	0	58.4
C4	2222	929	753	260	280	41.8	33.9	11.7	12.6
C5	4704	2769	1935	0	0	58.9	41.1	0	0
C6	5029	2337	2692	0	0	46.5	53.5	0	0
C7	4266	3840	426	0	0	90.0	10.0	0	0
C8	2079	1519	560	0	0	73.1	26.9	0	0
C9	1526	356	1170	0	0	23.3	76.7	0	0
C10	3653	1090	2561	0	2	29.8	70.1	0	0.1
C11	2330	1798	532	0	0	77.2	22.8	0	0
C12	7092	5233	0	1707	152	73.8	0	24.1	2.1
C13	4614	3777	837	0	0	81.9	18.1	0	0

## Data Availability

The data presented in this study are available on request from the corresponding author. The data will become publicly available with the publication of [49,75].
